# Supplementary material for: Assembling bacterial puzzles: piecing together functions into microbial pathways
Source: NAR Genom Bioinform. 2024 Aug 24;6(3):lqae109. doi: 10.1093/nargab/lqae109 (PMC11344244; doi:10.1093/nargab/lqae109)
Supplement: lqae109_Supplemental_Files [file lqae109_supplemental_files.zip › pamfun_SOM.pdf]

Supplementary materials for ‘Assembling bacterial puzzles: piecing together functions into microbial pathways’

Henri Chung, Iddo Friedberg, Yana Bromberg

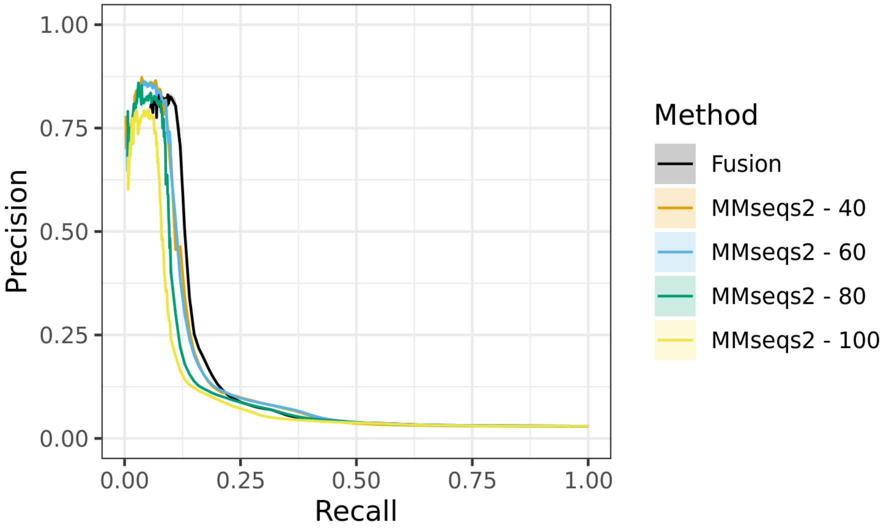

Figure S1: **Precision-Recall Curve.** Precision Recall Curve comparison between *fusion* and MMseqs2 profiles. *Fusion* profiles recall more pathway protein pairs than MMseqs2 profiles at comparable levels of precision across multiple bit-score thresholds (40-100, colored lines). For both profiles, there is a steep decrease in precision at recall values above 0.15.

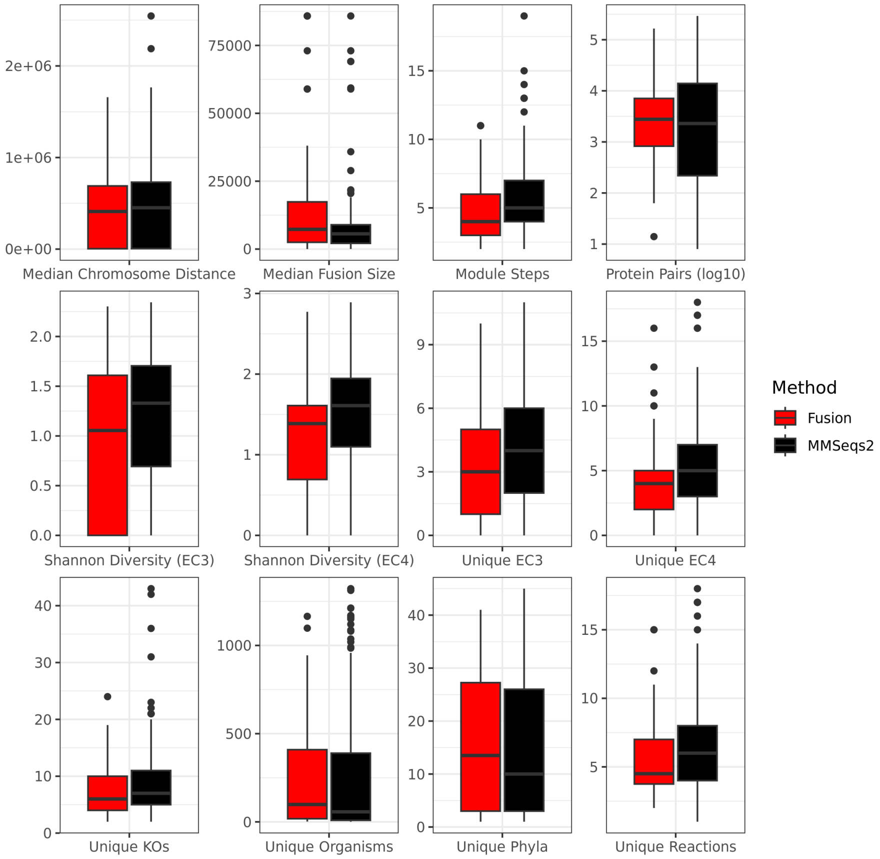

Figure S2: **Comparison of Module Variables.** Fusion and MMSeq2 annotations of KEGG modules differed in (1) median chromosome distance between proteins; (2) median *fusion* cluster number of constituent enzymes; (3) The number of reaction steps in the module; (4) number of protein pairs; Shannon diversity of (5) EC3 and (6) EC4 annotations; number of unique (7) EC3 and (8) EC4 annotations; (9) number of unique KOs; (10) number of unique organisms; (11) number of unique phyla for modules with a greater percentage of total links correctly predicted by *fusion* and MMseqs2 - 40 profiles; and (12) The number of unique chemical reactions in each module.

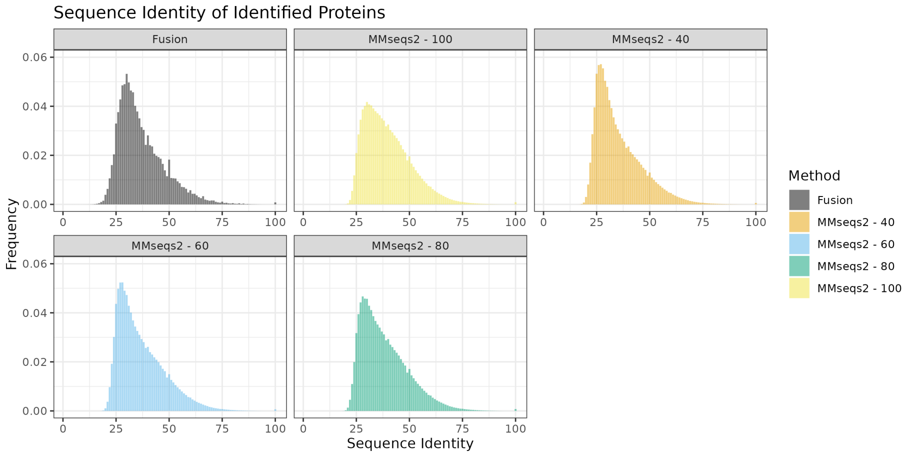

Figure S3: **Sequence Identity Comparison.** Distribution of local pairwise sequence identities for proteins group by each profiling method. The median pairwise sequence identity of proteins in *fusion* profiles was 34.7%. For MMseqs2 profiles, the median pairwise sequence identity was 32.9%, 34.6%, 36.4%, and 38.2% for bit-score thresholds of 40, 60, 80, and 100, respectively.

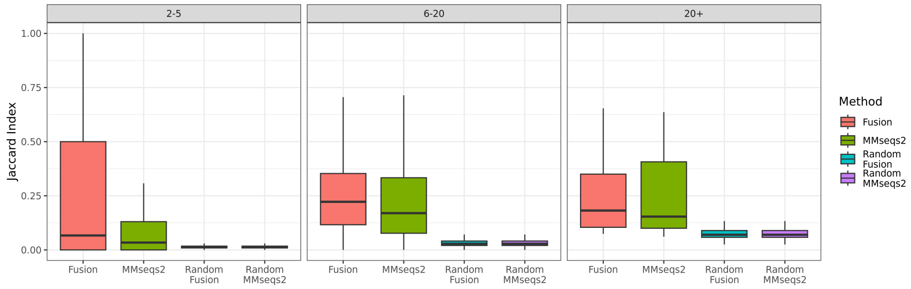

Figure S4: **Maximal Jaccard Similarities.** Comparison of the maximal Jaccard similarity for 62,378 organism-specific KEGG modules against clusters generated by *fusion* and MMseqs2 and their random comparisons. Modules separated into bins of 2-5, 6-20, and 20+ proteins. For all bin sizes, *fusion* clusters achieved a higher maximal Jaccard similarity with KEGG modules.

Table S1. Fusion and MMseqs2 Module Comparison. A table comparing the number of *fusion* and MMseqs2 profile predictions grouped by KEGG module. The largest fold change in module subclass was for M00063 CMP-KDO biosynthesis (42), M00615 Nitrate assimilation (39.6), M00847 Heme biosynthesis (33.8) and M00064 ADP-L-glycero-D-manno-heptose biosynthesis (28.4). MMseqs2 profiles predicted zero pairwise interactions for M00066 Lactosylceramide biosynthesis, M00112 Tocopherol/tocotorienol biosynthesis, and M00643.

Module ID	Module Class	MMseqs2	Fusion	Total	Fold Change
M00066	P:Lipid metabolism	0	3	14	Inf
M00112	P:Metabolism of cofactors and vitamins	0	44	385	Inf
M00643	S:Gene set	0	15	141	Inf
M00063	P:Glycan metabolism	42	1765	9479	42
M00615	S:Module Set	10	396	4656	39.6
M00847	P:Metabolism of cofactors and vitamins	4	135	1145	33.8
M00064	P:Glycan metabolism	27	767	7598	28.4
M00135	P:Amino acid metabolism	7	156	1574	22.3
M00967	P:Biosynthesis of other secondary metabolites	1	21	150	21
M00616	S:Module Set	21	317	3868	15.1
M00896	P:Metabolism of cofactors and vitamins	9	121	1013	13.4
M00551	P:Xenobiotics biodegradation	6	74	938	12.3
M00555	P:Amino acid metabolism	23	260	1978	11.3
M00881	P:Metabolism of cofactors and vitamins	95	1066	4228	11.2
M00151	P:Energy metabolism	60	633	6219	10.6
M00156	P:Energy metabolism	55	565	6831	10.3
M00745	S:Gene set	3	27	373	9
M00554	P:Carbohydrate metabolism	34	304	2163	8.94
M00377	P:Energy metabolism	59	392	6077	6.64
M00175	P:Energy metabolism	108	699	6480	6.47
M00899	P:Metabolism of cofactors and vitamins	109	666	5116	6.11
M00879	P:Amino acid metabolism	31	176	1087	5.68
M00133	P:Amino acid metabolism	75	372	6414	4.96
M00569	P:Xenobiotics biodegradation	112	497	9462	4.44
M00176	P:Energy metabolism	346	1313	23292	3.79
M00598	P:Energy metabolism	8	25	192	3.12
M00023	P:Amino acid metabolism	4966	14078	52430	2.83
M00079	P:Glycan metabolism	12	33	96	2.75
M00804	P:Energy metabolism	6	16	128	2.67
M00027	P:Amino acid metabolism	147	367	2873	2.5
M00153	P:Energy metabolism	211	518	2500	2.45
M00956	P:Amino acid metabolism	8	19	180	2.38
M00623	P:Xenobiotics biodegradation	14	31	451	2.21
M00880	P:Metabolism of cofactors and vitamins	1784	3765	19145	2.11
M00632	P:Carbohydrate metabolism	433	884	14253	2.04
M00087	P:Lipid metabolism	1732	3408	31911	1.97
M00565	P:Carbohydrate metabolism	199	390	6942	1.96
M00014	P:Carbohydrate metabolism	29	54	512	1.86
M00957	P:Amino acid metabolism	145	242	2398	1.67
M00579	P:Energy metabolism	272	443	5392	1.63
M00528	P:Energy metabolism	225	355	1417	1.58
M00026	P:Amino acid metabolism	14683	21159	99062	1.44
M00174	P:Energy metabolism	227	296	1801	1.3
M00015	P:Amino acid metabolism	1181	1420	11888	1.2
M00125	P:Metabolism of cofactors and vitamins	3369	4035	20327	1.2
M00088	P:Lipid metabolism	49	55	562	1.12
M00345	P:Energy metabolism	243	270	3347	1.11
M00375	P:Energy metabolism	15	16	53	1.07
M00020	P:Amino acid metabolism	572	593	9351	1.04
M00597	P:Energy metabolism	46	48	197	1.04
M00570	P:Amino acid metabolism	7081	7298	70330	1.03
M00095	P:Biosynthesis of terpenoids and polyketides	369	371	2219	1.01

Table S2. Unxpected KEGG modules in the marine metagenome. We identified KEGG modules in marine metagenomes, filtering to modules where all constituent components are present and which were not directly attributable to a specific organism in a sample by Kaiju annotation. We have compiled these 'unexpected' complete modules into a single list.

Module Class	Module subClass	ModuleID
P:Carbohydrate metabolism	Other carbohydrate metabolism	M00014
P:Lipid metabolism	P:Lipid metabolism	M00088
P:Energy metabolism	ATP synthesis	M00154
P:Carbohydrate metabolism	Central carbohydrate metabolism	M00308
P:Energy metabolism	Methane metabolism	M00357
P:Amino acid metabolism	Cysteine and methionine metabolism	M00368
P:Carbohydrate metabolism	Other carbohydrate metabolism	M00532
P:Amino acid metabolism	Branched-chain amino acid metabolism	M00535
P:Metabolism of cofactors and vitamins	Cofactor and vitamin metabolism	M00573
P:Metabolism of cofactors and vitamins	Cofactor and vitamin metabolism	M00577
P:Energy metabolism	Carbon fixation	M00620
S:Gene set	Drug resistance	M00725
S:Gene set	Drug resistance	M00726
S:Gene set	Drug resistance	M00730
P:Carbohydrate metabolism	Other carbohydrate metabolism	M00741
S:Gene set	Drug resistance	M00744
P:Carbohydrate metabolism	Other carbohydrate metabolism	M00761
P:Biosynthesis of other secondary metabolites	Biosynthesis of other antibiotics	M00785
P:Biosynthesis of terpenoids and polyketides	Terpenoid backbone biosynthesis	M00849
S:Gene set	Pathogenicity	M00857
P:Metabolism of cofactors and vitamins	Cofactor and vitamin metabolism	M00884
P:Metabolism of cofactors and vitamins	Cofactor and vitamin metabolism	M00897
P:Metabolism of cofactors and vitamins	Cofactor and vitamin metabolism	M00913
P:Metabolism of cofactors and vitamins	Cofactor and vitamin metabolism	M00914
P:Biosynthesis of other secondary metabolites	Biosynthesis of phytochemical compounds	M00953

SuppData1.txt

Supplementary Data 1. Balanced Fusion Data Assemblies. Genome assembly accession IDs of organisms used in the Balanced Dataset. A complete data set of 8,906 bacterial organisms were filtered and reduced to a set of organisms representing 96% of the taxonomic diversity using Treemer. The final dataset used for profile construction consisted of 1,393 bacterial organisms.

SuppData2.csv

Supplementary Data 2. Metagenome Interaction Predictions. Listed are pairs of co-occurring protein functions, identified by their EC4 numbers. Co-occurring functions were filtered to a Jaccard similarity $\geq$ 0.90 and were not found to co-occur in any of the identified taxa. These interacting pairs are putative marine metagenome emergent interactions between bacteria (or members of undetected bacterial genomes).

SuppData3.csv

Supplementary Data 3. Kaiju Annotations. A table of Kaiju-identified bacterial taxa annotated to each marine metagenome sample. Columns indicate the metagenome sample id, the KEGG ID, KEGG organism code, assembly accession, and taxon id of each taxa, along with the count of reads assigned to that taxon, the percent of the total reads in the sample to which the taxon contributes, and the phylum level taxonomic class.

SuppData4.csv

Supplementary Data 4. Metagenome Predictions by EC number. Predicted marine metagenome pathways, defined by member EC numbers. Each line indicates a group of EC numbers clustered together based on the Jaccard similarity of their metagenome profiles.

SuppData5.csv

Supplementary Data 5. Metagenome Predictions by *fusions* clusters. Predicted marine metagenome pathways, defined by member *fusion* clusters. Each cluster represents a putative marine metagenome pathway. Pathway components are listed by constituent *fusion* clusters, which contain proteins with both known and unknown functions. Fusion is available from `"https://figshare.com/articles/dataset/fusion_proteins_to_functions_tsv_gz/21599544"`
